# Transport properties in CFTR−/− knockout piglets suggest normal airway surface liquid pH and enhanced amiloride-sensitive Na^+^ absorption

**DOI:** 10.1007/s00424-020-02440-y

**Published:** 2020-07-25

**Authors:** Roberta Benedetto, Raquel Centeio, Jiraporn Ousingsawat, Rainer Schreiber, Melanie Janda, Karl Kunzelmann

**Affiliations:** 1 Institut für Physiologie, Universität Regensburg, Universitätsstraße 31, D-93053 Regensburg, Germany; 2 Institute of Molecular Animal Breeding and Biotechnology, Ludwig-Maximilians-Universität München, Munich, Germany

**Keywords:** CFTR, Chloride secretion, Bicarbonate transport, CFTR−/− piglets, Airways, Intestinal epithelium, Cystic fibrosis

## Abstract

Previous analysis of CFTR-knockout (CFTR−/−) in piglets has provided important insights into the pathology of cystic fibrosis. However, controversies exist as to the true contribution of CFTR to the pH balance in airways and intestine. We therefore compared ion transport properties in newborn wild-type (CFTR+/+) and CFTR-knockout (CFTR−/− piglets). Tracheas of CFTR−/− piglets demonstrated typical cartilage malformations and muscle cell bundles. CFTR−/− airway epithelial cells showed enhanced lipid peroxidation, suggesting inflammation early in life. CFTR was mainly expressed in airway submucosal glands and was absent in lungs of CFTR−/− piglets, while expression of TMEM16A was uncompromised. mRNA levels for TMEM16A, TMEM16F, and αβγENaC were unchanged in CFTR−/− airways, while mRNA for SLC26A9 appeared reduced. CFTR was undetectable in epithelial cells of CFTR−/− airways and intestine. Small intestinal epithelium of CFTR−/− piglets showed mucus accumulation. Secretion of both electrolytes and mucus was activated by stimulation with prostaglandin E2 and ATP in the intestine of CFTR+/+, but not of CFTR−/− animals. pH was measured inside small bronchi using a pH microelectrode and revealed no difference between CFTR+/+ and CFTR−/− piglets. Intracellular pH in porcine airway epithelial cells revealed only a small contribution of CFTR to bicarbonate secretion, which was absent in cells from CFTR−/− piglets. In contrast to earlier reports, our data suggest a minor impact of CFTR on ASL pH. In contrast, enhanced amiloride-sensitive Na^+^ absorption may contribute to lung pathology in CFTR−/− piglets, along with a compromised CFTR- and TMEM16A-dependent Cl^−^ transport.

## Introduction

In contrast to mouse CFTR-knockout models for cystic fibrosis (CF) [13], the CF pig model accurately reproduces human CF lung pathology [8, 38]. Pezzulo et al. found that the airway surface liquid (ASL) pH was more acidic in CF pigs when compared with wild-type littermates. They further showed that lower pH in CF animals inhibited the antimicrobial activity of ASL [40]. This correlated with impaired bacterial killing and disrupted mucociliary transport due to adhesive mucus, while enhanced airway Na^+^ absorption was  not detected [18, 51]. Hoegger et al. demonstrated an abnormal mucociliary transport in CF airways even under submerged conditions, thus questioning the role of surface dehydration in CF lung disease [18]. A large number of additional studies demonstrated the role of Cl^−^ and HCO_3_^−^ transport for proper mucociliary clearance [1, 9, 21, 24, 25]. A pathogenic concept was established in which CF lung disease starts with reduced HCO_3_^−^ secretion caused by impaired CFTR function. As a result, ASL acidification, impaired defense, inflammation, and mucus hypersecretion/adhesion attenuate mucociliary clearance and cause CF.

However, Schultz and coworkers assessed airway pH using an optical device connected to a fluorometer and measured pH directly in lungs of children with or without CF. They did not find evidence for acidic pH in the airways of CF children [47]. Moreover, Hug and Bridges did not find a change in intracellular pH of porcine submucosal gland cells upon cAMP-dependent stimulation of CFTR [19], and also Kim et al. reported an only limited contribution of CFTR to bicarbonate transport [27]. This is surprising given the reported impact of CFTR on ASL pH [40]. Due to these controversies, we aimed to analyze intrabronchial pH in piglets and intracellular pH in airway epithelial cells from CFTR+/+ and CFTR−/− piglets.

Chen et al. did not did detect an increase in transepithelial Na^+^ transport or enhanced liquid absorption in airways of CFTR−/− pigs, although an increase in amiloride-sensitive voltage was detected [8]. These results were in sharp contrast to the pathogenic concept of Na^+^ hyperabsorption in CF. According to this, enhanced electrolyte absorption driven by amiloride-sensitive Na^+^ absorption leads to airway dehydration and mucus plugging [6, 14, 33]. As a consequence, airway mucus plugging and impaired mucociliary clearance will lead to subsequent chronic bacterial infections. Because of these obvious contradictions, we re-examined ion transport in airways from CFTR+/+ and CFTR−/− piglets.

A CF pig model similar to the one described above was generated earlier by Klymiuk and coworkers [28]. CF-typical abnormalities were detected in the intestine, respiratory tract, and other epithelial organs, closely resembling human CF and the alterations observed in another CF pig model [36, 41]. Because functional parameters were not assessed in this CF pig model, we examined pH and ion transport in airways and the intestine. In contrast to [8, 40], we were unable to detect a lower pH in small airways. In contrast, we found evidence for enhanced Na^+^ absorption. The present data support the concept of airway dehydration and hyperabsorption as the primary cause for impaired mucus clearance in CF [17].

## Methods

### Generation of CFTR−/− piglets and tissue preparation

Generation and breeding of CFTR+/+ and CFTR−/− littermate piglets were performed as described earlier [28]. In brief, CFTR−/− piglets have been generated by inactivating the CFTR gene in primary porcine cells by sequential targeting, using modified bacterial artificial chromosome vectors. The cells were then used to generate homozygous CFTR-mutant piglets by somatic cell nuclear transfer. All animal procedures were performed according to the German Animal Welfare Act with permission of the local regulatory authority of the LMU Munich. Piglets were euthanized within the first 24 h after birth under Ketamine (Ursotamin®, Serumwerk Bernburg, Germany) and Azaperone (Stresnil®, Elanco Animal Health, Bad Homburg, Germany) anesthesia by intracardiac injection of T61® (Intervet, Unterschleissheim, Germany).

### Intrabronchial pH measurements

Intrabronchial pH was assessed with a micro-pH electrode (Orion™ 9810BN, Thermo Scientific™, Schwerte, Germany). The electrode requires as little as 500 nl liquid to reliably determine pH values. Immediately after sacrificing the animals, lungs were removed, sliced and electrodes were placed 5 mm deep into cross-sectionally opened small airways with diameters between 1.5–3 mm. At least 10 pH readings were taken, and a mean value was determined for each airway. The spherical 1.3 mm pH electrode tip was polished and did not penetrate airway epithelial cells with a diameter of about 20 μM. The pH values obtained through these measurements represent the pH of airway surface liquid and mucus present in small airways.

### Primary airway epithelial cell cultures

Porcine bronchial epithelial (PBE) cells were harvested from isolated bronchi of wild-type (CFTR+/+) and CFTR-knockout (CFTR−/−) piglets as previously described for human lung cells [10]. In brief, primary cells were collected in bronchial epithelial cell basal medium supplemented with bovine pituitary extract, insulin, hydrocortisone, retinoic acid, transferrin, triiodothyronine, epinephrine, and human epidermal growth factor (Lonza, Basel, Switzerland). Cells were cultured using Rho kinase and dual SMAD signaling inhibition in the absence of a feeder-cell layer and were re-differentiated on permeable supports.

### HE and Alcian blue staining, lipid peroxidation

Histological analysis was performed as described earlier [2]. Tissues were fixed in 4% paraformaldehyde, picric acid, and sucrose in PBS and were washed in methanol before embedding in paraffin. Sections were stained with Alcian blue and assessed by light microscopy. A 4-HNE staining was used to analyze membrane lipid peroxidation, as described earlier [46]. Thirty images of 4 CFTR−/− animals and 6 images of 3 CFTR+/+ animals were examined. Sections were analyzed using an Axiovert200 microscope equipped with AxioCam ICc 1 and ApoTome (Zeiss, Oberkochen, Germany).

### Immunocytochemistry

Paraffin-embedded sections (5 μM) were blocked with 5% bovine serum albumin (BSA) and 0.04% Triton X-100 in PBS for 30 min. TMEM16A was detected using P79 anti-TMEM16A (1:300; Davids Biotechnology, Regensburg, Germany). CFTR was detected using mouse monoclonal anti-CFTR antibody #596, kindly provided by the North American CF foundation. The CFTR antibody was used at a dilution of 1:3000. Immunofluorescence was detected with an Axiovert 200 and analyzed using AxioVision software (AxioVs40; V 4.8.2.0; Zeiss, Jena, Germany).

### RT-PCR

For RT-PCR total RNA from lung tissue or porcine primary cells was isolated using NucleoSpin RNA II columns (Macherey-Nagel, Düren, Germany). Total RNA (1 μg/50 μl reaction) was reverse-transcribed using random primer (Promega, Mannheim, Germany) and M-MLV Reverse Transcriptase, RNase H Minus (Promega, Mannheim, Germany). Semiquantitative RT-PCR was used to determine levels of expression for each transport protein. To that end, mRNA from at least *n* = 3 CFTR+/+ and *n* = 3 CFTR−/− animals was analyzed in a larger number of reactions. Each RT-PCR reaction contained sense and antisense primers (0.5 μM) (Table 1), 0.5 μl cDNA, and GoTaq Polymerase (Promega, Mannheim, Germany). After 2 min at 95 °C, cDNA was amplified (35 cycles for target sequence and 30 cycles for the reference GAPDH) for 30 s at 95 °C, 30 s at 56 °C, and 1 min at 72 °C. PCR products were visualized by loading on peqGREEN (Peqlab; Düsseldorf, Germany) containing agarose gels and analyzed using ImageJ.

**Table 1:** Primers for RT-PCR

CFTR	Forward: 5′- AACCTGAACAAGTTTGATGAAG Reverse: 5′- CAGAACAATGCAGAATGAGATG	480 bp
TMEM16A	Forward: 5′- CGTCATCATCAACCTGGTGG Reverse: 5′- CCAGGCGGATCTCAATGATG	587 bp
TMEM16F	Forward: 5′- GGAGTTTTGGAAGAGGCGC Reverse: 5′- CAATAAACTGGATCTCCTGGG	579 bp
SLC26A4	Forward: 5′- CACCATCGACGGGAATCAG Reverse: 5′- GCAAGTAAACACCCAGATAAC	672 bp
SLC26A9	Forward: 5′- CATACTCCCTCACCCTCTTC Reverse: 5′- CTCTCATTGGTGGCATTGTTG	433 bp
SCNN1A	Forward: 5′- CTGCAACAACACCACCATCC Reverse: 5′- GGAGTTGTACTTGTACAGGTC	313 bp
SCNN1B	Forward: 5′- GTGACAACACCAACACCCAC Reverse: 5′- GAGAAGATGTTGGTGGCCTG	598 bp
SCNN1G	Forward: 5′- GCGCCCACTATCAAGGAGC Reverse: 5′- CCTTGCCCGTCTCACCTTG	454 bp
GAPDH	Forward: 5′- CATCGGGCGCCTGGTCAC Reverse: 5′- CTCCTGGAAGATGGTGATGG	199 bp

### In vitro perfusion of piglet jejunum ex vivo

The methods for in vitro perfusion of excised intestinal section were explained in previous publications [2]. In brief, piglets were euthanized, and excised intestinal sections were placed immediately in ice-cold Ringer solution (mmol/l 145 NaCl, 0.4 KH2PO4, 1.6 K2HPO, 4.6 D-glucose, 1 MgCl2, 1.3 Ca^2+^ gluconate, pH 7.4). Sections were carefully flushed to remove residual luminal contents. Tissues were mounted into an Ussing chamber insert with a circular aperture of 0.785 mm^2^. Luminal and basolateral sides of the epithelium were perfused continuously at a rate of 5 ml/min. Solutions were heated to 37 °C, using a water jacket. ENaC and cyclooxygenase were inhibited by amiloride (10 μM) and indomethacin (10 μM), respectively. IBMX (100 μM) and forskolin (2 μM) (IF) were used to measure CFTR-dependent Cl^−^ secretion in jejunal tissue. Carbachol (CCH; 100 μM) was used to activate Ca^2+^-dependent Cl^−^ transport. Experiments were carried out under open circuit conditions. Data were collected continuously using PowerLab (AD Instruments, Spechbach, Germany). Values for transepithelial voltages (*V*_te_) were referred to the serosal side of the epithelium. Transepithelial resistances (*R*_te_) were determined by applying short (1 s) current pulses (ΔI = 0.5 μA). *R*_te_ and equivalent short circuit currents (Isc) were calculated according to Ohm’s law (*R*_te_ = Δ*V*_te_/ΔI, Isc = *V*_te_/*R*_te_). To assess mucus secretion, intestinal segments were mounted and perfused basolaterally in a custom-designed perfusion chamber, with HCO_3_^−^ containing Ringer solution at 37 °C and bubbling with 95% O_2_/5% CO_2_, as described earlier (Fig. 3e) [2]. The lumen was perfused with glucose-free, HCO_3_^−^ Ringer solution at a rate of approximately 0.5 ml/min. The system was let run for 30 min to remove residual luminal contents. Mucus release was stimulated by apical PGE_2_ and ATP. Apical perfusates were collected in 3-min intervals, and DTT was added to dissolve mucus. Mucin content of the luminal perfusates was analyzed using Periodic acid-Schiff (PAS) and absorbance assays. Values were normalized to the weight of the jejunal section.

### Intracellular pH measurements

Porcine primary cells were incubated in Ringer solution (mmol/l NaCl 118.75; KH2PO4 0.4; K2HPO4 1.6; glucose 5; MgCl2 1; Ca-gluconate 1.3; probenecid 2.5; Na-gluconate 25) containing 2 μM BCECF-AM (Life Technologies GmbH, Darmstadt, Germany) and 0.02% Pluronic (Life Technologies) for 60 min at 20 °C. For intracellular pH measurements, cells were mounted in a cell chamber and perfused at 37 °C with HCO_3_^−^/CO_2_ containing Ringer solution (mmol/l, NaCl 118.75; KH2PO4 0.4; K2HPO4 1.6; glucose 5; MgSO4 1; Ca-gluconate 1.3; probenecid 2.5; NaHCO3 25; bubbled with 95% O_2_/5% CO_2_). Pendrin activity was measured by the initial slope of pH increase after applying Cl^−^-free HCO_3_^−^/CO_2_ solution (Na-gluconate 118.75; KH2PO4 0.4; K2HPO4 1.6; glucose 5; MgSO4 1; Ca-gluconate 1.3; probenecid 2.5; NaHCO3 25; bubbled with 95% O_2_/5% CO_2_) and Cl^−^-free iodide solution (Na-gluconate 118.75; KH2PO4 0.4; K2HPO4 1.6; glucose 5; MgSO4 1; Ca-gluconate 1.3; probenecid 2.5; NaHCO3 25; bubbled with 95% O_2_/5% CO_2_). For pH calibration, cells were perfused with buffers of variable pHs between 6.5 and 8.5 containing 105 mosmol/l KCl, 1 mmol/l MgCl2, 30 mmol/l HEPES, and 5 μmol/l nigericin. Excitation wavelengths of 440 and 490 nm were used, and emission intensity at 535 nm was recorded using a high speed polychromator system and a CoolSnap HQ camera (Visitron Systems, Puchheim, Germany). Control of experiment, imaging acquisition, and data analysis were done with the software package Meta-Fluor (Universal imaging, USA).

### *Patch clamping*

Cells were grown on coated glass coverslips for patch clamp experiments. Patch pipettes were filled with a cytosolic-like solution containing in mM, KCl 30; K-gluconate 95; NaH_2_PO_4_ 1.2; Na_2_HPO_4_ 4.8; EGTA 1; Ca-gluconate 0.758; MgCl_2_ 1.03; D-glucose 5; ATP 3; and pH 7.2. The Ca^2+^ activity was 0.1 μM. Coverslips were mounted in a perfused bath chamber on the stage of an inverted microscope (IM35, Zeiss) and kept at 37 °C. The bath was perfused continuously with Ringer solution at a rate of 8 ml/min. Patch clamp experiments were performed in the whole cell configuration. Patch pipettes had an input resistance of 4–6 MΩ when filled with cytosolic like solution. Currents were corrected for serial resistance. The access conductance was monitored continuously and was 60–140 nS. Currents (voltage clamp) and voltages (current clamp) were recorded using a patch clamp amplifier (EPC 7, List Medical Electronics, Darmstadt, Germany), the LIH1600 interface and PULSE software (HEKA, Lambrecht, Germany), as well as the Chart software (AD Instruments, Spechbach, Germany). Data were stored continuously on a computer hard disc and analyzed using PULSE software. At regular intervals, the membrane voltage (*V*c) was clamped in steps of 20 mV from − 100 to + 100 mV from a holding voltage of − 100 mV.

### Transepithelial Ussing chamber recordings

Filter-grown airway epithelial cells were measured under open or short circuit conditions as detailed in previous reports [4, 29]. In brief, PBE cells were grown on Millipore filters in an air-liquid interface (ALI) in Advanced DMEM/F12 media (Thermo Fisher Scientific, USA) supplemented with 0.5 μg/mL hydrocortisone, 100 nM triiodothyronine, and 0.5 μg/mL epinephrine (all from Sigma-Aldrich, Missouri, USA); 0.25 μg/mL human epidermal growth factor (PeproTech, UK); 100 nM TTNPB (Cayman, USA); and 50 nM A83-01 (Tocris Bioscience, Bristol, UK) for 14–21 days. For the first week of ALI, 500 nM A83-01 was supplemented, and for the second week, 10 μM of DAPT (Tocris Bioscience, Bristol, UK) was added.

### Materials and statistical analysis

All compounds used were of highest available grade of purity. Data are reported as mean ± SEM. The data were symmetrically distributed as continuous (not ordinal) data. Student’s *t* test (for paired or unpaired samples as appropriate) and ANOVA were used for statistical analysis. A *p* value < 0.05 was accepted as significant difference. The total number of CFTR+/+ and CFTR−/− animals was 13 and 14, respectively. For each experimental series, the number of animals used and the number of measurements/assays/reactions are provided (number of animals/number of experiments).

## Results

### Airway abnormalities in newborn CFTR−/− piglets

**Fig. 1:**
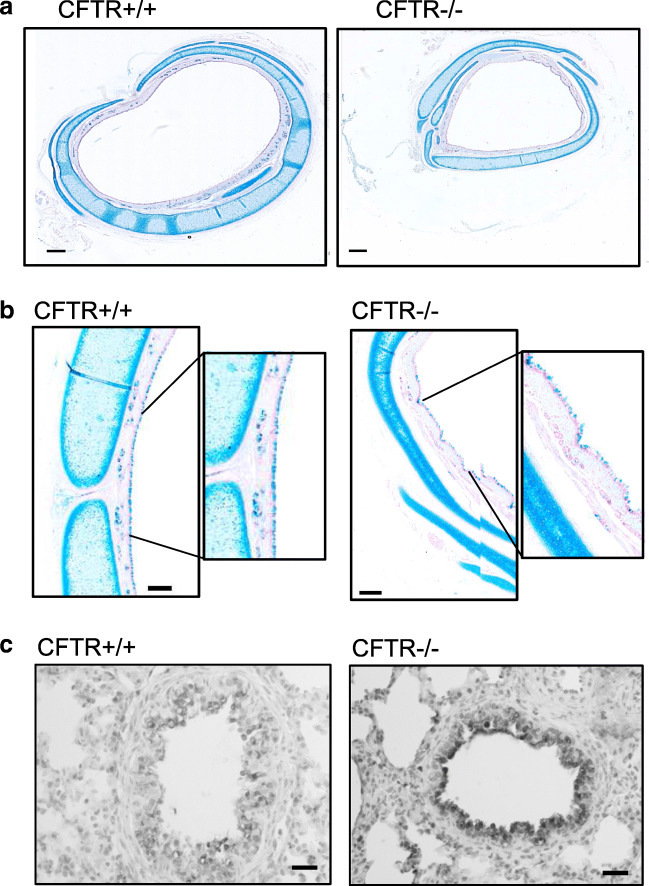
Structural changes in airways from CFTR−/− piglets. **a** Comparison of cross-sections of tracheas from CFTR+/+ (*n* = 2) and CFTR−/− (*n* = 2) newborn piglets. Bars = 500 μM. **b** Higher magnification showing defective cartilage structure in muscle bundles in CFTR−/− tracheas. Bars = 80 μM. **c** Small airways from CFTR+/+ (*n* = 3) and CFTR−/− (*n* = 3) newborn piglets. Dark precipitation is caused by 4-hydroxy-2-nonenal (4-HNE) staining, indicating membrane lipid peroxidation of airway epithelial cells in CFTR−/− tracheas. Typically, 34–40 images were examined in each series. Bars = 50 μM

Generation and basic pathological properties of CFTR−/− piglets have been described in a previous report [28]. Due to local ethical regulations and 100% penetrance of the meconium ileus, piglets needed to be sacrificed 24 h after birth. Discontinued tracheal cartilage rings and altered orientation of smooth muscle cell bundles described by Klymiuk et al. [28] were also observed in the present study (Fig. 1a,b). Similar to the CFTR−/− piglets described by Rogers et al. [41], we also did not observe accumulation of mucus in small or larger airways of CFTR−/− piglets, and no evidence was found for mucus plugging. Rogers et al. did not detect evidence for airway/lung inflammation in newborn CFTR−/− piglets, arguing against early lung inflammation in young infants before bacterial colonization [41]. We examined whether lipid peroxidation as an early inflammatory marker is present in small airways of newborn CFTR−/− piglets. This would provide hints as to an early lung inflammation in CF. Notably, in 3 out of 4 piglets examined, evidence for lipid peroxidation of airway epithelial cells was detected using 4-HNE staining, while none of the CFTR+/+ piglets showed significant staining (Fig. 1c).

### Expression of CFTR and TMEM16A

**Fig. 2:**
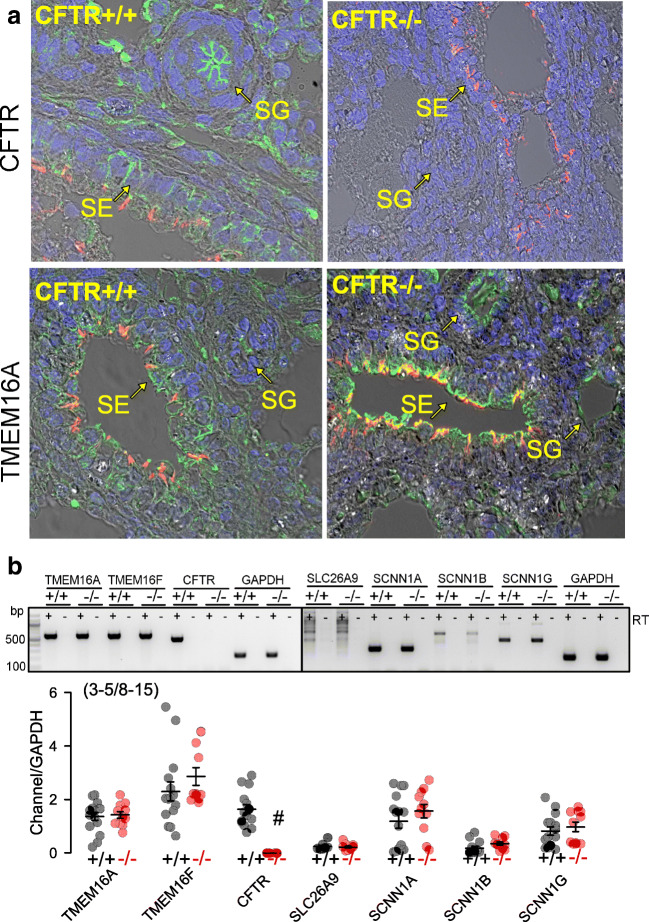
Expression of ion channels in porcine airway epithelial cells. a Expression of CFTR and TMEM16A (green fluorescence) in airways from newborn CFTR+/+ and CFTR−/− piglets. In WT lungs, CFTR is expressed predominately in submucosal glands (SG). Some expression is found in the surface epithelium (SE). No expression of CFTR is detected in CFTR−/− lungs. In CFTR+/+, TMEM16A is expressed in SE and SG at low level but appears somewhat upregulated in lungs of CFTR−/− animals. Cilia (acetylated tubulin, red) and nuclei (DAPI). About 20–25 images of each *n* = 3 CFTR−/− and CFTR+/+ animals were examined. **b** Analysis of mRNA expression for TMEM16A, TMEM16F, CFTR, SLC26A9, and αβγENaC (SCNN1A, SCNN1B, SCNN1G) in airway epithelial cells from newborn CFTR+/+ and CFTR−/− piglets. Mean ± SEM (number of animals/number of reactions). ^#^Significant difference when compared to CFTR+/+ (*p* < 0.05, unpaired *t*-test)

We examined the expression of CFTR and the Ca^2+^-activated Cl^−^ channel TMEM16A using immunohistochemistry. In CFTR+/+ airways, CFTR was well expressed in airway submucosal glands and surface epithelium, while no CFTR was detected in CFTR−/− animals (Fig. 2a). TMEM16A was found in submucosal glands and surface epithelium of CFTR+/+ piglets and was also present in airways of CFTR−/− animals. mRNA expression for TMEM16A, TMEM16F, SLC26A9, and αβγENaC were found to be similar in CFTR+/+ and CFTR−/− airway epithelial cells, while CFTR-mRNA was completely absent in CFTR−/− (Fig. 2b).

### Lack of intestinal chloride and mucus secretion in CFTR−/− piglets

**Fig. 3:**
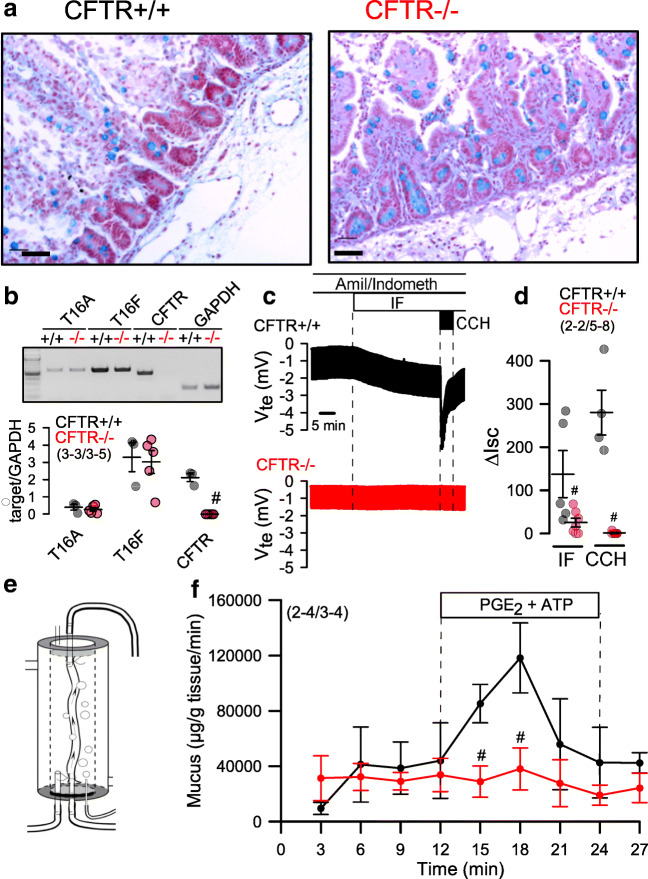
Lack of intestinal fluid and mucus transport in CFTR−/− piglets. **a** Histology and Alcian blue staining of intestinal epithelium from CFTR+/+ and CFTR−/− piglets. Bars = 100 μM. Representative stainings from *n* = 2 CFTR+/+ and CFTR−/−, respectively, with 22–23 images being analyzed. **b** Expression of TMEM16A, TMEM16F, and CFTR in intestinal epithelial cells from CFTR+/+ and CFTR−/− piglets as detected by RT-PCR. **c** Original Ussing chamber recordings of transepithelial voltages (open circuit) from freshly isolated jejunal epithelium obtained from CFTR+/+ (upper recoding; black) and CFTR−/− (lower recording; red) piglets. **d** Comparison of calculated short circuit currents activated by basolateral IBMX (100 μM) and forskolin (2 μM) (IF) or the muscarinic agonist carbachol (CCH, 100 μM). **e** Scheme for the perfusion of isolated CFTR+/+ and CFTR−/− jejunum ex vivo (left) and **f** time course for mucus release induced by luminal perfusion with PGE_2_ (1 μM) and ATP (100 μM). Mean ± SEM (number of animals/number of experiments or reactions). ^#^Significant difference when compared with CFTR+/+ (*p* < 0.05, ANOVA)

Klymiuk et al. described an intestinal phenotype in CFTR−/− piglets, resembling the severe intestinal phenotype observed in CF [28]. A similar phenotype was found in another CFTR−/− model described earlier [41]. In the present study, mucus accumulation was present in small intestinal crypts of CFTR−/− animals (Fig. 3a). Expression of CFTR-mRNA was absent in isolated crypt epithelial cells from CFTR−/− animals, while expression of TMEM16A or TMEM16F-mRNA was similar to that in CFTR+/+ animals (Fig. 3b). In Ussing chamber recordings of small intestinal sections of CFTR−/− piglets, we observed a complete absence of both cAMP-activated (IF) and Ca^2+^-activated (CCH) Cl^−^ transport (Fig. 3c,d). This result confirms earlier findings obtained in human intestinal biopsies, showing that Ca^2+^-dependent electrolyte secretion relies entirely on the presence of apical CFTR [34]. We reported earlier mouse intestinal mucus secretion by apical purinergic stimulation [2]. Using the same method of in vitro perfused intestinal loops (Fig. 3e), we found that ATP and PGE_2_-induced mucus secretion was completely absent in small intestine freshly removed from CFTR−/− animals (Fig. 3f). The results are in line with earlier findings in mouse, demonstrating the essential role of CFTR for intestinal mucus secretion [48].

### CFTR−/− piglets do not exhibit low bronchiolar pH

**Fig. 4:**
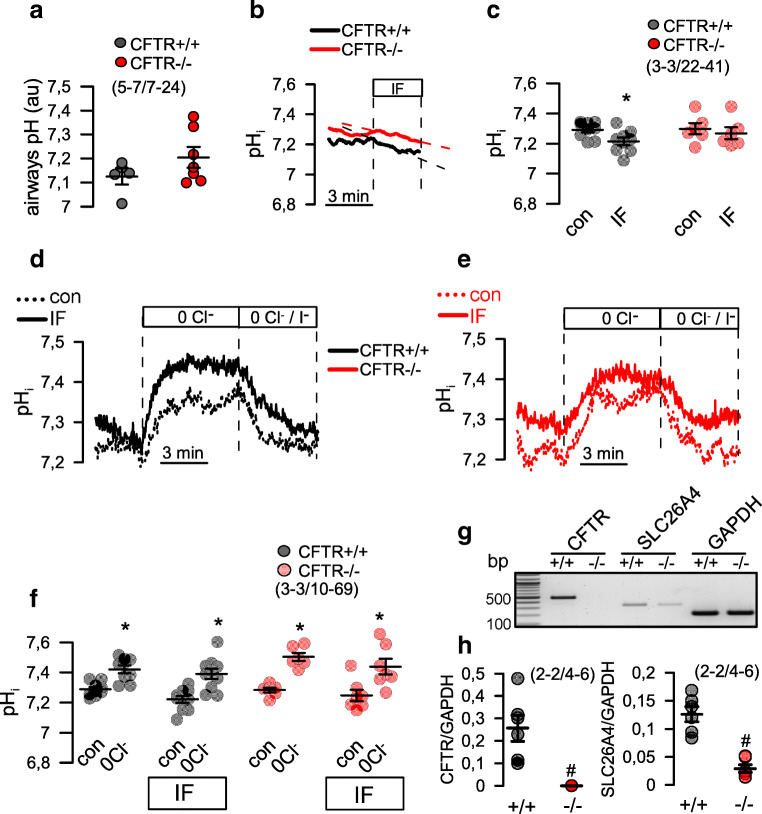
Limited impact of CFTR on pH regulation in piglet airways. **a** Intraluminal pH measured in bronchioles of CFTR+/+ and CFTR−/− piglets. Per animal, *n* = 7–24 measurements were taken and pooled. **b,c** Intracellular pH (pH_i_) measured in airway epithelial cells isolated from newborn CFTR+/+ and CFTR−/− piglets. Stimulation with IF had little but significant effects on pH_i_ of CFTR+/+ cells. **d,e** Original recordings of pH_i_ in airway epithelial cells from CFTR+/+ and CFTR−/− piglets. Effect of removal of extracellular Cl^−^ (0 Cl^−^) and addition of I^−^ (0 Cl^−^/I^−^) on pH_i_ in the absence or presence of IF. **f** Summaries of the experiments shown in (**d,e**). *n* = 3 CFTR+/+ and CFTR−/− animals. Per animal, *n* = 10–69 measurements were taken and pooled. **g,h** Expression of CFTR and SLC26A4 in airway epithelial cells from CFTR+/+ and CFTR−/− piglets as detected by RT-PCR. Mean ± SEM (number of animals/number of measurements). *Significant effect of IF and 0 Cl^−^, respectively. ^#^significant difference when compared with CFTR+/+, respectively (*p* < 0.05, ANOVA)

Evidence has been presented for reduced tracheal surface pH in CF pigs [40]. In the present CF porcine model, we assessed intrabronchial pH using a micro-pH electrode. The electrode had a very small tip diameter allowing pH measurements directly in small airways. pH was assessed in peripheral airways of different sections in each lung. We detected a mean value for pH value of about 7.1 in airways of CFTR+/+ piglets, which corresponded well to the pH value detected in vivo in CFTR+/+ piglets reported by Pezzulo and coworkers [40]. However, in contrast to this study, we did not detect a lower pH value in airways of CFTR−/− piglets (Fig. 4a).

### Limited contribution of CFTR to regulation of intracellular pH

This result prompted us to measure intracellular pH in primary bronchial airway epithelial cells. We found that intracellular pH was similar in cells from CFTR+/+ and CFTR−/− piglets and was between 7.2 and 7.3. pH was only slightly acidified upon stimulation with IF in CFTR+/+ but not in CFTR−/− cells, suggesting an only limited contribution of CFTR to bicarbonate secretion (Fig. 4b,c). Removal of extracellular Cl^−^ caused an intracellular alkalization in both CFTR+/+ and CFTR−/− cells, while IF did not further increase intracellular alkalization (Fig. 4d–f). Moreover, we found expression of pendrin mRNA being lower in CFTR−/−, when compared with CFTR+/+ cells (Fig. 4g,h). Taken together, the data suggest a limited contribution of CFTR to the regulation of intracellular pH in porcine airway epithelial cells. As reported earlier, other transport proteins, such as pendrin, might be more important for pH regulation in airway epithelial cells [11, 15].

### Enhanced Na^+^ absorption and defective cAMP and Ca^2+^-dependent Cl^−^ secretion in CFTR−/− airway epithelial cells

**Fig. 5:**
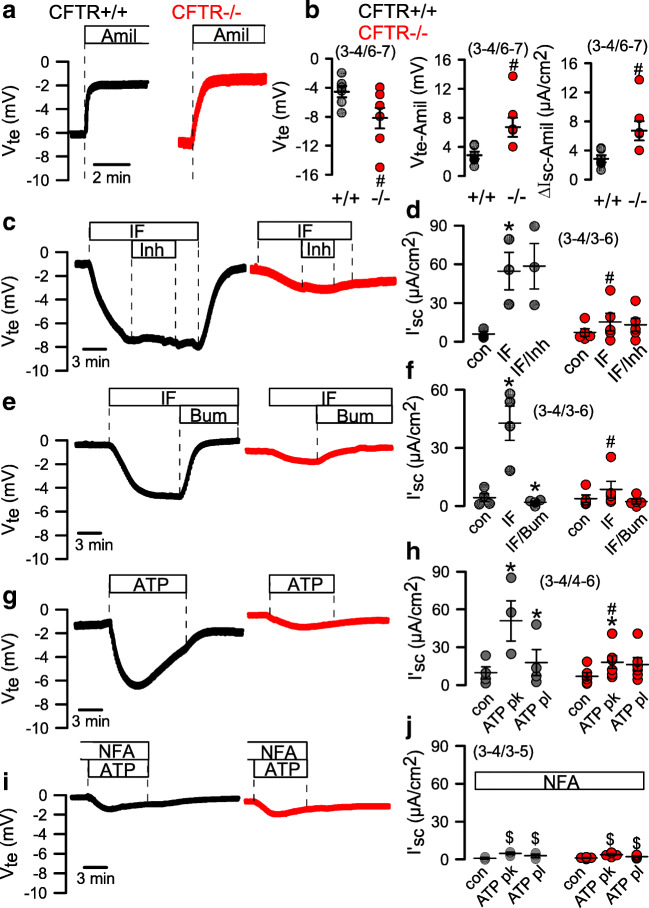
Comparison of ion transport in airway epithelial cells from CFTR+/+ and CFTR−/− piglets. **a** Open circuit Ussing chamber measurements on polarized grown airway epithelial cells from CFTR+/+ and CFTR−/− piglets in the absence of CO_2_/HCO_3_^−^. Effects of amiloride (10 μM). **b** Comparison of voltage deflections and calculated equivalent short currents (Isc). **c,d** Effect of IBMX/forskolin (IF 100/2 μM) and CFTRinh172 (30 μM) on transepithelial voltages and Isc. **e,f** Effect of IF and bumetanide (100 μM) on transepithelial voltages and Isc. **g,h** Effect of luminal ATP (100 μM) on transepithelial voltages and Isc (pk, peak current; pl, plateau current). **i,j** Effect of luminal ATP (100 μM) in the presence of niflumic acid (NFA; 10 μM) on transepithelial voltages and Isc. Mean ± SEM (number of animals/number of measurements). *Significant effects of IF, Bum, or ATP (*p* < 0.05, paired *t*-test). ^#^Significant difference when compared with CFTR+/+ (*p* < 0.05, unpaired *t*-test). ^$^Significant difference when compared with the absence of NFA (*p* < 0.05, unpaired *t*-test)

Cl^−^ and Na^+^ transport was analyzed in the absence of bicarbonate in polarized grown airway epithelial cells from CFTR+/+ and CFTR−/− piglets. We first used open circuit conditions in a perfused micro-Ussing chamber, to analyze transepithelial voltages (*V*_te_). We found slightly but significantly augmented *V*_te_ in CFTR−/− monolayers and enhanced inhibition of *V*_te_ by amiloride (Δ*V*_te-Amil_). The calculated equivalent short circuit currents inhibited by apical amiloride (Δ*I*_sc-Amil_) was enhanced in CFTR−/− epithelia (Fig. 5a,b). The data may indicate enhanced Na^+^ absorption in CF airways. In contrast, lumen negative voltage deflections induced by cAMP-dependent stimulation (IF) were largely reduced in CFTR−/− cells, indicating a lack of CFTR expression (Fig. 5c). Surprisingly, luminal application of the CFTR inhibitor CFTRinh172 did not inhibit IF-induced Cl^−^ secretion in CFTR+/+ airways. However, this has also been observed earlier in naïve tissues or mucus producing monolayers. In contrast, the NKCC1 inhibitor bumetanide (Bum, basolateral) completely abolished Cl^−^ transport in CFTR+/+ monolayers and also blocked the small transport observed in CFTR−/− cells (Fig. 5c–f).

Notably, Ca^2+^-dependent Cl^−^ secretion induced by luminal ATP demonstrated a similar kinetic as IF-induced secretion and was also inhibited in CFTR−/− cells (Fig. 5g,h). ATP-activated secretion in CFTR+/+ cells was fully inhibited by the TMEM16A blocker niflumic acid. These results and previous reports suggest that receptor-mediated Ca^2+^-activated Cl^−^ secretion in porcine and human airways essentially occurs through CFTR, which, however, requires the function of TMEM16A [5, 37]. Although still incompletely understood, expression and/or function of TMEM16A appears essential to transmit signals from P2Y_2_ - receptors to CFTR [4, 31].

**Fig. 6:**
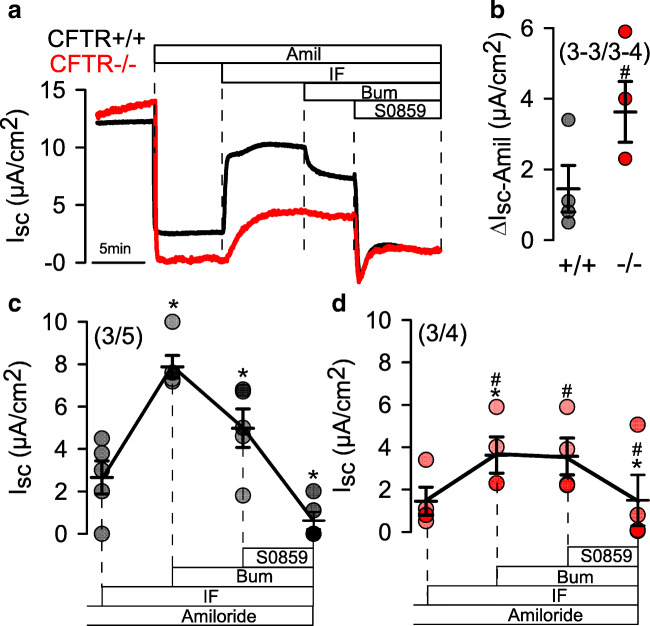
Cl^−^ and HCO_3_^−^ transport in airway epithelial cells from CFTR+/+ and CFTR−/− piglets. **a,b** Short circuit Ussing chamber measurements on polarized grown airway epithelial cells from CFTR+/+ and CFTR−/− piglets in the presence of 5% CO_2_ and 25 mM HCO_3_^−^. Pronounced inhibition of Isc by amiloride (10 μM). **c** Summaries for the activation and inhibition of Isc by subsequent addition of IF, Bum, and S0859 (S, 30 μM). Mean ± SEM (number of animals/number of filters). *Significant effects of IF, Bum, or S (*p* < 0.05, paired *t*-test). Difference when compared with the absence of IF or when compared with CFTR+/+. ^#^Significant difference when compared to CFTR+/+ (p < 0.05, unpaired t-test).^

In contrast to an earlier report, the present data suggest enhanced Na^+^ absorption in airways of CFTR−/− piglets [8]. We performed additional transepithelial measurements under real short circuit conditions and in the presence of HCO_3_^−^ (Fig. 6a). Short circuit currents were strongly inhibited by amiloride and were larger in monolayers from CFTR−/− piglets (Fig. 6a,b). In the presence of amiloride, IF-induced Isc was significantly reduced in CFTR−/− monolayers but was still well detectable (Fig. 6c,d). Bumetanide inhibited the short circuit current in CFTR+/+, but not CFTR−/− monolayers. The remaining portion of IF-activated Isc was blocked by S0859, an inhibitor of the basolateral sodium bicarbonate cotransporter (NBC) (Fig. 6c,d). Taken together, the data suggest an upregulated Na^+^ absorption by the epithelial Na^+^ channel ENaC, a lack of cAMP-activated Cl^−^ secretion in CFTR−/− cells, and a significant cAMP-activated HCO_3_^−^ transport that is reduced but still detectable in CFTR−/− airways.

### Ion transport was also assessed in patch clamp experiments

**Fig. 7:**
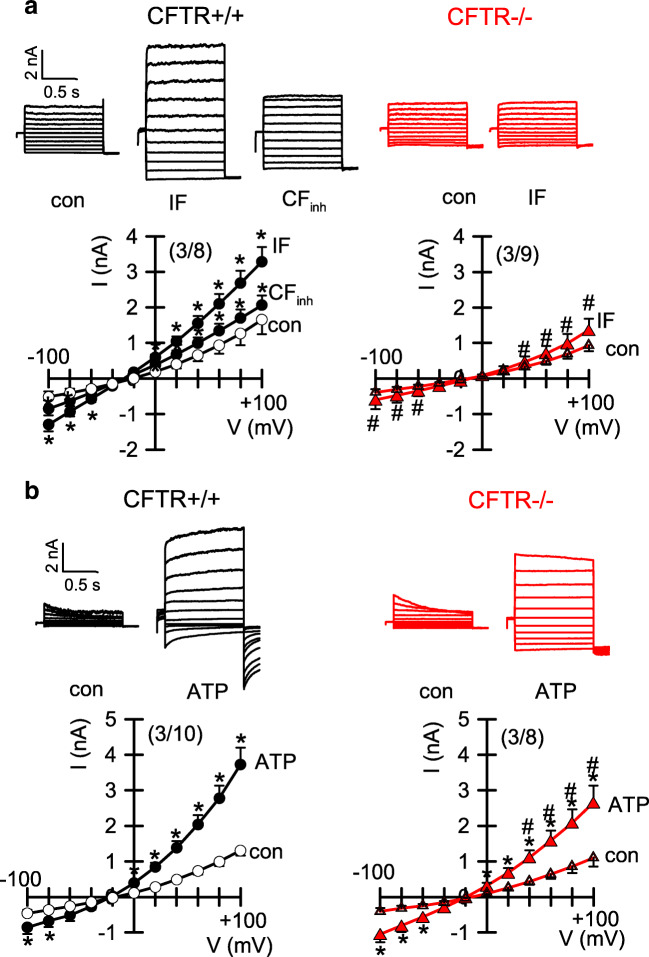
Ion currents in airway epithelial cells from CFTR+/+ and CFTR−/− piglets. **a,b** Ion current overlays and current/voltage relationships obtained in whole cell patch clamp recordings with airway epithelial cells from CFTR+/+ and CFTR−/− piglets. **a** IBMX/forskolin (IF, 100/2 μM) activated whole cell currents were only detected in cells from CFTR+/+ animals. Currents were inhibited by 30 μM CFTRinh172 (CFinh). **b** ATP (100 μM) activated whole cells were reduced in CFTR−/− cells. Mean ± SEM (number of animals/number of cells). *Significant effects of IF, ATP, or CFinh (*p* < 0.05, paired *t*-test). ^#^Significant difference when compared with CFTR+/+ (*p* < 0.05, unpaired *t*-test)

Here, we found activation of CFTR whole cell currents by stimulation with IF in CFTR+/+ cells. IF-activated currents were inhibited by CFTRinh172 (Fig. 7a). In contrast, no currents were activated by IF in CFTR−/− cells. Ca^2+^-activated whole cell currents were activated by ATP and were found to be reduced in CFTR−/− cells (Fig. 7b). Thus, both cAMP-dependent and Ca^2+^−activated secretions are compromised in the airways of CFTR-knockout piglets. Moreover, in contrast to polarized grown epithelial cells and naive airways, the strict CFTR-dependence of ATP-activated Cl- currents is lost in non-polarized cells. We conclude that the direct Cl- transporting capacity of TMEM16A in piglet and human airways is probably negligible.

## Discussion

The present data compare functional aspects of airway and intestinal epithelial cells from newborn WT and CFTR-knockout piglets [28]. Pulmonary oxidative stress response and abnormal bioactive lipids have been demonstrated in CF lung disease [20, 45], suggesting early pulmonary inflammation [42, 52]. Lipid peroxidation was also detected in the present study in the airways of newborn CFTR−/− piglets, providing evidence for an early pulmonary inflammation in the absence of bacterial colonization (Fig. 1). CF epithelial cells were shown earlier to have a compromised anti-oxidant defense by superoxide dismutase [43]. Finally, some CFTR-knockout mouse models did show signs of lung/airway inflammation even in the absence of mucus obstruction [53]. Thus, early intrinsic inflammation without mucus plugging or bacterial infection may initiate CF lung disease [45].

While intrabronchial mucus accumulation or mucus plugging was not observed in airways of newborn CF piglets, accumulation of mucus in small intestinal crypts was obvious and comparable to earlier observations [28, 41]. In vitro perfusion of the small intestine from CFTR−/− piglets with luminal ATP could not induce mucus release (Fig. 3). In contrast, stimulation of CFTR+/+ jejunum by luminal ATP induced mucus secretion similar to mouse small and large intestine [2]. Notably, stimulation by prostaglandin E2 or ATP alone did not induce mucus secretion [2, 7]. These data correspond very well to the goblet cell dysfunction detected earlier in CF intestine, which is likely to contribute to intestinal obstruction and inflammation in CF [32].

**Fig. 8:**
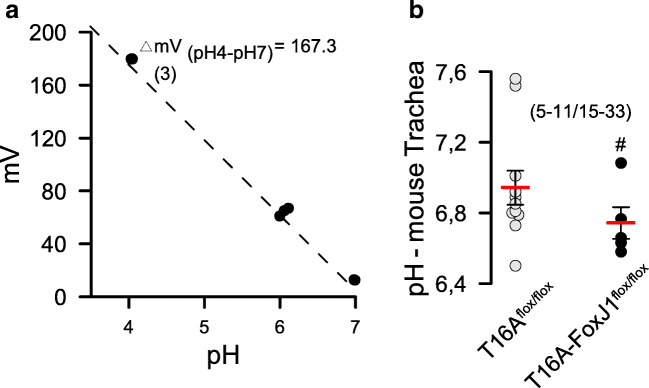
Calibration curve and pH values in mouse trachea. **a** Calibration curve for the microelectrode used in airway pH measurements (*n* = 3 measurements each). **b** pH measured in tracheas of T16A^flox/flox^ wild-type mice and mice with a knockout of TMEM16A in ciliated airway epithelial cells (T16A^flox/flox^-FoxJ1Cre). Mean ± SEM (number of animals/number of pooled measurements). ^#^Significant difference when compared with T16A^flox/flox^ (*p* < 0.05, unpaired *t*-test)

In contrast to a previous report on another porcine CFTR-knockout model [40], we did not find evidence for a reduced pH in small airways of CFTR−/− piglets (Fig. 4). Correspondingly, we found no difference in intracellular pH of CF and non-CF porcine airway epithelial cells under resting conditions and little impact of CFTR on intracellular pH. Bicarbonate transport was reduced in CF airway epithelia but nevertheless was still detectable (Fig. 6c,d). These data along with previous studies suggest that pendrin-dependent HCO_3_^−^ secretion may be more important in controlling ASL pH [11, 27, 50]. We are confident that our intrabronchial microelectrode measurements were correct, as the standard curve obtained with this electrode provided reliable and reproducible pH readings (Fig. 8). Moreover, control measurements of mouse intratracheal pH showed a value of 6.98 ± 0.16 (*n* = 33), which corresponded well with other intratracheal in vivo measurements described earlier [23, 49]. Moreover, intraluminal measurements in mouse intestine showed pH values of 3.4 for stomach and 6.5 for colon, which is similar to earlier results [35]. Therefore, the pH values obtained in piglet airways probably reflect the true situation. For comparison, we also measured intratracheal pH in mice with an airway-selective knockout of TMEM16A (T16A^flox/flox^-FoxJ1Cre) [4]. Notably, the intratracheal pH in these animals was significantly lower when compared with WT animals (Fig. 8b). Thus, TMEM16A is likely to contribute to the regulation of ASL pH. In fact, TMEM16A was shown earlier to be permeable for bicarbonate and has been discussed as a potential channel for bicarbonate release in the airway epithelium [12, 16, 26]. Despite the evidence for acidic airways in T16A^flox/flox^-FoxJ1Cre mice, the animals did not show any lung pathology [4], again questioning the role of acidic pH for lung pathology in CF.

Pezzulo et al. reported lower pH values of the airway surface liquid (ASL) covering the tracheal epithelium of newborn CFTR−/− piglets. It is possible that the pH of tracheal and bronchial ASL may be different. However, regarding the CF lung disease, intrabronchial pH values are probably more relevant to the disease. Saint-Criq et al. performed long-term ASL pH measurements on fully differentiated primary human airway epithelial cells under very stable conditions [44]. ASL pH was found not to be different between non-CF and CF under resting (non-stimulated) conditions, but was enhanced in non-CF after stimulation with forskolin. Schulz and collaborators measured ASL pH in vivo in children using a luminescence technology integrated with fiber optic probes. They demonstrated that ASL pH in children with CF is similar to that of children without CF [47]. Our pH measurements directly in the lumen of small bronchi support these findings, as they revealed similar pH values in CF and non-CF piglets.

Because ASL thickness is just a few microns, accurate pH assessments are difficult. Probably any pH measurement technique has its own drawbacks, whether it is done by pH sensitive foils (CO_2_ alterations), optical pH probes (light scattering by mucoid environment), in vitro measurements (incomplete differentiation, lack of submucosal glands), or pH electrode measurements (accurate location of electrodes). In addition, anesthesia compromises breathing patterns and circulation, while immediate measurement postmortem affects ion transport and CO_2_. More work is required to solidify the concept of airway acidification as the cause for defective host defense in CF [49, 50]. At any rate, the pathogenic concept of airway acidification as the initiator of CF lung disease is also seriously questioned by the recent findings that patients with a loss of TMEM16A-function also lack CFTR function [39]. Yet, these patients do not develop any lung disease. Similarly, mice that lack of TMEM16A in ciliated epithelial cells also lack CFTR function [3, 4]. Again, these mice do not develop a lung disease, although they even show a lower intratracheal pH (Fig. 8b). In contrast, in both open and short circuited Ussing chamber measurements and in the absence or presence of bicarbonate, we found evidence for enhanced amiloride-sensitive Na^+^ transport in primary CF airway epithelia (Figs. 5 and 6). This is in contrast to data reported from another CF porcine model [22], but is in line with many previous measurements obtained ex vivo in human respiratory tissues*,* primary airway epithelial cells, and CF intestine [30]. Taken together, the present data question the pathogenic role of defective CFTR-dependent ion transport and acidic pH in CF, but rather suggest that intrinsic airway inflammation along with Na^+^ hyperabsorption is the cause for CF lung disease.
